# Videoscope-Assisted Minimally Invasive Surgery (VMIS) for Bone Regeneration around Teeth and Implants: A Literature Review and Technique Update

**DOI:** 10.3390/dj6030030

**Published:** 2018-07-06

**Authors:** Stephen K. Harrel

**Affiliations:** Department of Periodontology, Texas A&M College of Dentistry, Dallas, TX 75246, USA; skharrel@gmail.com; Tel.: +1-214-352-5304

**Keywords:** minimally invasive, videoscope, periodontal surgery, bone regeneration, bone grafts, biologics

## Abstract

Background—The literature related to minimally invasive periodontal surgery is reviewed. This includes the original minimally invasive surgery (MIS) procedure for bone regeneration, the modification of MIS for the minimally invasive surgery technique (MIST) and modified MIST (M-MIST) procedures, and the introduction of the videoscope for oral surgical procedures and the ability to perform videoscope-assisted minimally invasive surgery (VMIS). The evolution from MIS through MIST to the current VMIS is reviewed. The results from studies of each of these methods are reported. Conclusion—The use of small incisions that produce minimal trauma and preserve most of the blood supply to the periodontal and peri-implant tissues results in improved regenerative outcomes, minimal to absent negative esthetic outcomes, and little or no patient discomfort. Minimally invasive procedures are a reliable method to regenerate periodontal tissues.

## 1. Literature Review

Periodontal minimally invasive surgery for bone regeneration, termed MIS, was first described by Harrel and Rees in 1995 [1]. The MIS technique was further described in multiple publications from 1995 through 2000 [2,3,4,5]. The concepts of minimally invasive surgery for bone regeneration consist of the use of much smaller incisions than those traditionally used for periodontal bone grafting, the maintenance of as much of the blood supply as possible to aid in regeneration and to minimize patient discomfort, and the primary closure of the surgical incisions. At the time MIS was introduced, the visualization of the root surfaces and bony lesions for debridement and placement of demineralized freeze-dried bone allograft (DFDBA) bone grafting material was performed with surgical telescopes (3X to 5X magnification). Because of the challenges of visualization, at this initial stage of MIS surgical technique development, very small buccal and lingual flaps were used. The use of both a buccal and a lingual flap was necessary to allow for adequate visualization of the defect with the telescopes. 

The published results of MIS showed excellent improvement in pocket probing depth and attachment level. Multiple case series showed improvements in pocket probing depths of 3 to 5 mm with most post-surgical pocket depths measuring less than 4 mm [2,3,4]. The long-term post-surgical regenerative results were considered to be equal and, in many cases, superior to the results that were achieved with traditional periodontal bone grafting techniques using a large-flap approach. The patients usually indicated that they had minimal discomfort when MIS was used, but this was not formally measured at that time. However, with the early MIS approach, there was soft tissue recession of one plus mm, which is only slightly less than the amount of recession resulting from traditional surgical approaches. 

Harrel and Wilson performed a study using the previously described MIS approach with the addition of enamel matrix derivative (EMD) [6,7]. The flaps, visualization technique, and bone graft with DFDBA were the same as originally described, but, in addition, the manufacturers’ recommendation for the use of EMD was followed, and EMD was also added to the DFDBA. Part of the manufacturers’ recommendation for the use of EMD is root biomodification with EDTA. This was done for the stated purpose of removing the “smear layer” produced during root planing of the root surface and was designed to allow the EMD to be absorbed into the root surface. The one-year and five-year post-operative data from MIS with EMD were reported on 160 sites. This study showed that excellent results were obtained at one year and that the improvement was maintained for at least five years. The mean improvement in pocket probing depths was 3.50 mm, and the improvement in clinical attachment level (CAL) was 3.48 mm. These results were felt to be an improvement over the results obtained with traditional surgical techniques for bone regeneration. In addition, the post-surgical soft tissue height showed a small mean improvement. With traditional regenerative surgical approaches, a soft tissue loss (recession) of 1–2 mm was reported [8].

While the regenerative results from MIS were considered to be equivalent or improved compared to the regenerative improvements obtained from traditional regenerative surgical approaches, from a clinician and patient satisfaction point of view the results were considered a major improvement over past results. A significant positive feature was the lack of post-surgical recession. Up until these studies, some amount of recession had been reported for all regenerative procedures. The lack of esthetically compromising recession was a new finding.

In 2007, Cortellini and Tonetti published a modification of the original MIS procedure [9]. They termed their procedure the minimally invasive surgery technique (MIST). This procedure used an incision pattern very similar to MIS for access to the bony defect. The major modification of the MIS procedure was that MIST incorporated elements of the papilla preservation technique. These modifications involved the handling of the buccal flap and the suturing technique used to close the surgical site. Where the MIS procedures used a simple vertical mattress suture at the base of the papilla and the incisions were closed with finger pressure, the papilla preservation suturing used in MIST utilized overlapping sutures to approximate the edges of the incision. No bone graft was used in MIST, but EMD was placed in the lesion. Visualization for MIST was obtained using the surgical microscope. The reported results from MIST were very similar to those reported from MIS with EMD. Slightly more recession was noted following MIST (0.4 mm) than with MIS combined with EMD, but recession in both procedures was very minor to non-existent. Further reports by Cortellini and others also showed excellent results [10,11,12,13,14]. A latter change in the MIST procedure, termed modified MIST (M-MIST), was introduced that utilized only a buccal flap [12].

MIS and MIST used very similar incisions and tissue handling to achieve improvements in pocket probing depths and clinical attachment with minimal soft tissue changes. Both procedures yielded results that were equal to or improved over the regenerative results gained with traditional regenerative procedures such as bone grafts or guided tissue regeneration. Despite these excellent and consistent results from two separate research centers, minimally invasive small incision surgery did not become a routine approach for regeneration of bone in periodontal defects. While there are many factors that may have influenced the acceptance of minimally invasive techniques, the difficulty of visualizing the defect through the small openings was probably the leading factor. It can be extremely difficult to visualize the base of a defect or the accretions on a root surface with the technology used in the previously discussed studies, i.e., surgical loops or surgical microscope. 

## 2. Visualization Techniques

The original MIS procedure was performed with relatively high-magnification surgical telescopes with headlights. While this technology is a major improvement over direct visualization, the magnification available was inadequate for very small incisions, and it was frequently necessary to use longer incisions in order to visualize the defect. The use of a surgical microscope, as in MIST, improved the magnification available, but many surgeons find the bulkiness of the microscope to be a negative factor. If any patient movement occurs during a procedure, either the patient or the surgical microscope has to be meticulously repositioned. Another visualization method that was attempted for MIS was the use of a glass fiber flexible endoscope (Periovue) designed for non-surgical use. This device uses a water-filled environment to keep the lens of the endoscope clear. It was found that it was difficult to impossible to keep a surgical site filled with water, which made the use of this endoscope very difficult and time-consuming.

The goal for minimally invasive surgery is to regenerate lost periodontal supporting tissue utilizing the smallest possible incisions and flap openings. In addition, the maintenance or improvement of patient esthetics is a major goal. It became obvious that the visualization technology that had been used in MIS and MIST was inadequate to make the incisions smaller. Also, in order to attain the most esthetic end results, a visualization technology that could be easily used with a lingual approach, as opposed to a buccal approach or a buccal and lingual approach, was necessary. While both telescopes and surgical microscope can be used with a mirror to obtain visualization from the lingual, this extra step can be difficult in many areas of the mouth. The lack of visualization technology that was easy to use in the lingual approach was probably influential in the design of M-MIST where a buccal approach was used for access. An ideal visualization technology needs to be small enough to fit into a very small surgical access, easily maneuverable so the entire bony lesion and adjacent root surface can be easily observed, and capable of delivering good visualization using a single lingual flap.

**Figure 1:**
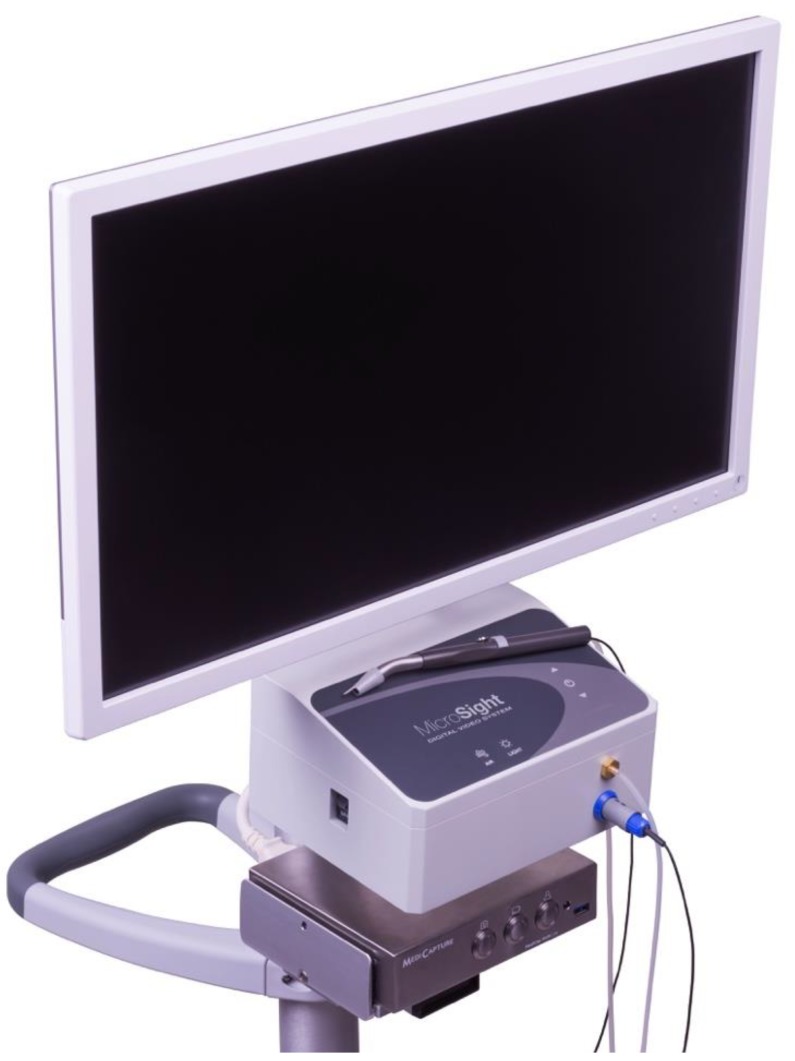
The videoscope designed for oral surgical procedures (MicroSight, Q-Optics, USA).

With these goals in mind, research was undertaken to create a new technology specifically designed for minimally invasive periodontal regenerative procedures. This research was made possible by a grant from the US National Institutes of Health (Bethesda, MD, USA). The result was a surgical videoscope designed for oral use. A videoscope is an instrument that has a very small digital camera that can be inserted directly into the surgical site. The image is transmitted from the camera to a monitor as an electronic signal. This differs from an endoscope in which a lens arrangement is inserted into the surgical site and the image is transmitted to an external camera through a fiber optic bundle. A videoscope is capable of a much clearer and true color picture of the surgical site than a fiber optic endoscope. The transmission of the image through the fiber optic bundle results in significant image degradation so that what is seen on the monitor is unclear and often has a false color. By transmitting an electronic signal, the camera in the videoscope has minimal optical distortion and the colors are more true to life. The surgical videoscope was first described in 2013 [15]. The currently used videoscope is pictured in Figure 1.

Another concern with any optical device placed in a surgical site is the obscuring of the optical lens by blood or surgical debris. When this occurs, the device must be removed from the surgical site, and the lens cleaned. Because the lens becomes obscured very quickly in the small surgical access used for MIS or MIST, it is impossible to use an optical device like a videoscope or endoscope without a method that keeps the lens clean. The dental endoscope used for non-surgical periodontal procedures addresses this problem with a constant flow of water. This is not feasible for a surgical procedure because the water is not adequately contained. In addition, the water adds a distortion to the image seen on the monitor. The videoscope used for minimally invasive surgery solves this problem by passing a stream of low-pressure air over the camera lens. This forms a vortex in front of the camera lens and effectively forces away debris form the lens and keeps the lens free from blood and other debris. The air pressure used is quite low and does not represent a danger of soft tissue emphysema [16].

## 3. Videoscope-Assisted Minimally Invasive Surgery (VMIS)

Following the development of the videoscope designed for use in minimally invasive periodontal regenerative surgery, a surgical procedure specifically designed to take advantage of the videoscope was developed. The MIS procedure was used as a starting point for the surgical technique that became known as Videoscope-Assisted Minimally Invasive Surgery (VMIS) [17,18,19,20]. The procedure is discussed in detail in the paragraphs below.

Indication—The most common situation in which to perform VMIS is for regeneration of bone loss in interproximal areas. Frequently, patients will initially present with generalized moderate to severe periodontitis. Following oral hygiene instruction and non-surgical root planing, most pocket probing depths may return to an acceptable level of 4 mm or less. However, there will frequently be isolated areas of bone loss where the pocket probing depth is unacceptably deep. The interproximal areas are the most frequent location of these deeper areas. With traditional surgery for regeneration, buccal and lingual incisions covering one or two teeth on either side of these isolated defects would be used. These longer incisions are made on periodontally healthy teeth and increase the probability of post-surgical recession and discomfort. With VMIS, incisions are made only in the area of the defect. It is unnecessary to extend the incisions to healthy areas for the sake of visualization. While the videoscope is used for many other surgical procedures beyond these isolated interproximal lesions, VMIS for this type of lesion will be described here.

**Figure 2:**
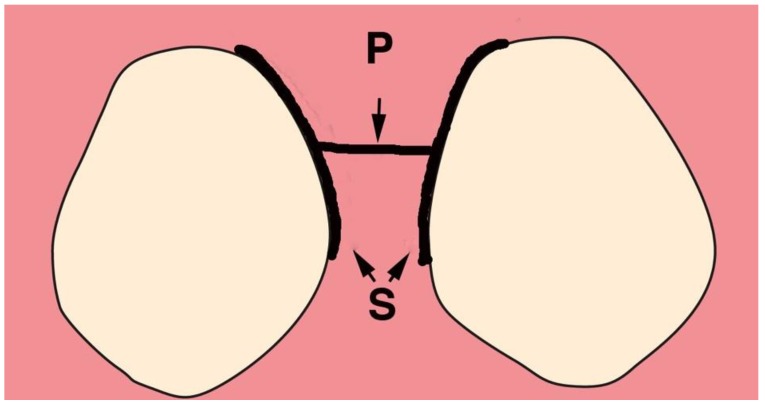
The incisions used for Videoscope-Assisted Minimally Invasive Surgery (VMIS). The incisions are made on the lingual aspect in the area of bone loss. Sulcular incisions (S) are made on the lingual aspect of the teeth adjacent to the area of interproximal bone loss. A split-thickness connecting incision is made at the base of the papilla (P).

Incisions—The incisions for VMIS are limited to the area of bone loss. In most cases, the mesial-distal length of the incision is no more than 6–8 mm. With the availability of the videoscope, it is not necessary to use a longer incision to obtain visualization. The incisions are shown in Figure 2. 

**Figure 3:**
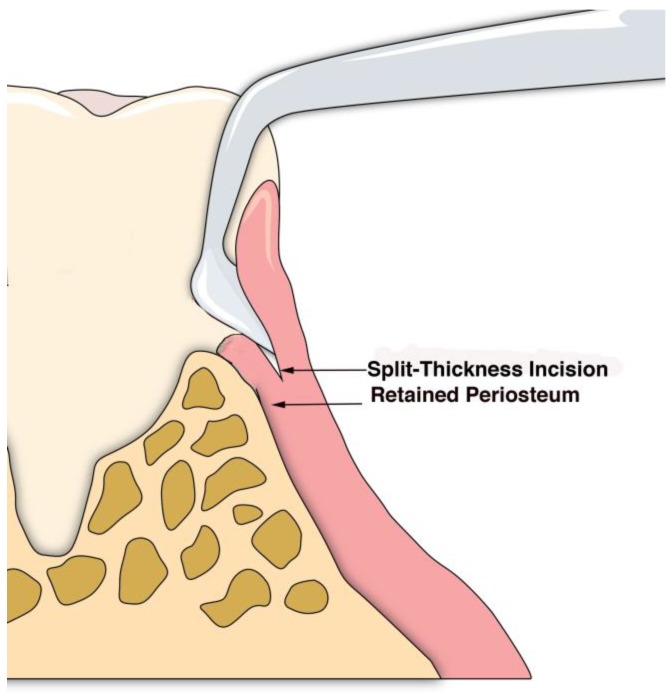
The split-thickness incisions at the base of the papilla (P in Figure 2) are designed to leave the periosteium intact. A periosteal elevator should not be used.

A sulcular incision (S in Figure 2) is made on the lingual aspect of the teeth on either side of the area of bone loss. When these incisions are made, the blade is placed against the root surface and inserted to the base of the defect. No tissue is removed with this incision. Unlike more traditional surgical approaches where the sulcus lining is removed or a “collar of tissue” is removed, the goal with VMIS incisions is to sever the granulation tissue only and to leave the rest of the tissue intact. Following the placement of the sulcular incisions, a connecting incision is made at the base of the papilla (P in Figure 2). This incision is made only to the crest of the bone. The goal is to retain as much of the periosteium on the bone as possible. Depending on the anatomy of the area, this may be the full extent of incisions that is necessary. If there is inadequate room to place the soft tissue retractor of the videoscope or if the bony defect cannot be adequately visualized, the papillary incision (P) can be extended apically as a split-thickness incision (Figure 3). Under no circumstance should a periosteal elevator be used to gain space. A periosteal elevator will pull the periosteium from the bone, which will greatly diminish the blood supply to the periodontal tissue. The use of a periosteal elevator is associated with a greater recession than that observed when a split-thickness approach is used.

**Figure 4:**
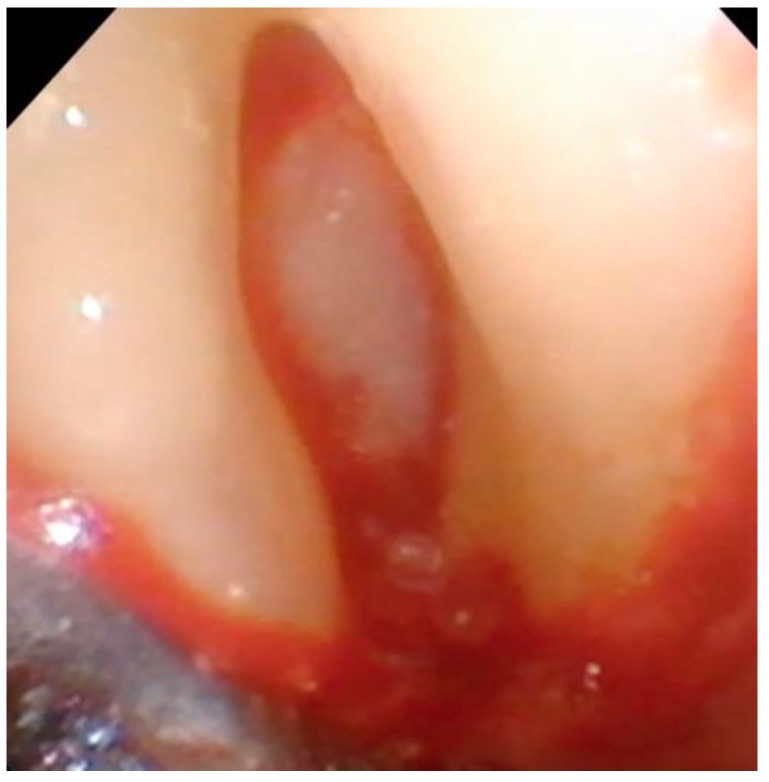
A furcation defect that has been fully debrided and EDTA used to remove any micro-islands of calculus. This site is ready for bone grafting and enamel matrix derivative (EMD) treatment.

Debridement—Once incisions and the small split-thickness lingual flap have been made, the retractor on the handpiece of the videoscope is inserted, and light pressure is placed on the flap so that the defect can be visualized. Generally, there will be a significant amount of granulation tissue in the defect which must be removed. This is usually performed with a Younger-Goode curette that has been reduced in size by about one-third from its original size. This curette is used in an action similar to using an instrument to “spoon” out caries from a tooth. This action is more suitable for the small flaps of VMIS than the traditional “scaling” action used with surgical curettes. Once the granulation tissue has been removed, the root surfaces can be debrided of accretions and roughness. This is performed with a combination of ultrasonic instrumentation and hand instruments. The videoscope is used throughout the debridement process. When an ultrasonic scaler is used, there will be some transient blurring of the videoscope image due to water on the lens, but the air over the lens clears the image in a matter of seconds when the ultrasonic scaler is turned off. After mechanical debridement of the roots, they are dried with gauze and thoroughly inspected with the videoscope. The 20 to 40× magnification of the videoscope often reveals “micro-islands” of calculus and anatomical roughness on the root surface. ^20^ These can be removed with the use of EDTA (Prefgel, Straumann, USA), recommended by the manufacturer for the biomodification of the root surface before using EMD. A fully debrided defect ready for bone grafting is shown in Figure 4.

Bone Graft—Following the use of EDTA to remove any remaining micro-islands of calculus, the defect is rinsed, and EMD is placed on the root surface. The remaining EMD is mixed with DFDBA or other bone grafting material and gently placed into the bony defect. The defect should not be overfilled, as that will potentially interfere with the ability to obtain primary closure of the soft tissue. Membranes are never used in VMIS. The incisions would have to be greatly expanded to accommodate a membrane, and the use of a membrane is unnecessary to achieve the results reported in the literature for VMIS. 

**Figure 5:**
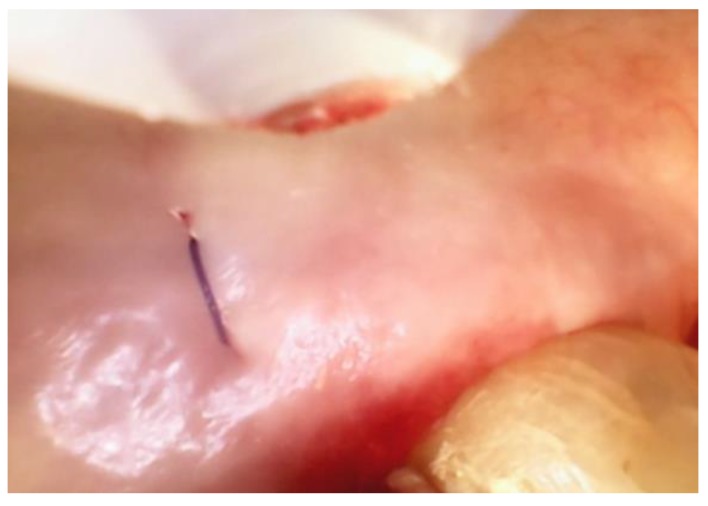
A Videoscope-Assisted Minimally Invasive Surgery (VMIS) palatal flap closed with a single vertical mattress suture at the base of the papilla. The edges of the incision in the papilla have been approximated with finger pressure only.

Suturing—A single vertical mattress suture placed at the base of the papilla is recommended for each site of VMIS. It is not necessary to use the papilla preservation suturing technique when the VMIS incision pattern is used. The suture should be placed in the thick area of tissue at the base of the papilla. No sutures are placed directly at the connecting incision between the two teeth. It has been determined that placing sutures in this thin tissue leads to greater post-operative recession. Once the single suture is placed at the base of the papilla, the incisions are primarily approximated by using finger pressure on a saline-soaked gauze. A closed surgical site is shown in Figure 5.

The surgical technique described above was utilized in a masked outcome-based case series of 30 patients and 110 sites. The surgical sites in this study were areas of pocket probing depth of 5mm or greater at six weeks post root planing that also had radiographic evidence of bone loss and no greater than a class-I furcation defect. The results from this case series were reported at six months, one year, and 3 to 5 years post-surgery [17,18,19,20]. Statistically significant improvements in pocket probing depth (3.8 mm), clinical attachment levels (4.16 mm), and soft tissue height (0.38 mm) were noted at all measurement intervals and were stable for the 3- to 5-year time period. All surgical sites were noted to be less than 4 mm at 5 years post-surgery, with a mean pocket probing depth of less than 3 mm.

Discomfort and patient satisfaction—Patient pain levels were evaluated on the day of surgery and at each follow-up appointment. Two patients indicated very slight discomfort on the day of surgery, one patient had slight discomfort one week post-surgery. With these exceptions, no patient reported any discomfort at any time following VMIS. All patients indicated satisfaction with the surgical procedure, satisfaction with the esthetic outcomes, and would have the procedure done again at another site if indicated. It should be noted that, in this long-term study, there was no mean recession in the surgical sites and there was a mean improvement in soft tissue height of nearly 0.5 mm, which contributed to patient satisfaction with esthetics. Both lack of recession and soft tissue improvement have not been reported with any other periodontal bone regenerative procedure.

## 4. VMIS for Peri-Implant Bone Loss

The VMIS procedure has been modified for regeneration in areas of bone loss on implants. The results from this new approach have been encouraging. A study is underway on this approach but has not been completed. 

The cause of bone loss around implants is unknown, and many different causes for bone loss around implants may exist. Clearly, because the implant interface with the bone is completely different from a natural tooth, it is unlikely that peri-implant bone loss is the same process that occurs in periodontal bone loss around natural teeth. Various factors have been suggested, including bacterial plaque, occlusion, and improper surgical placement of the implants. Wilson et al. showed that there were multiple particles of both titanium and cement embedded in the soft tissue surrounding many failed implants, and these particles were always surrounded by inflammatory cells [21]. The localized inflammatory response universally noted around these particles resembles a foreign body reaction seen with other materials. This phenomenon has led to the hypothesis that, in addition to excess cement, bone loss around implants may be associated with a foreign body reaction to titanium particles from the implant caused by corrosion or other stresses on the implant surface. The use of the VMIS procedure for treating implant bone loss is specifically aimed at removing any particles of cement or titanium that may be embedded in the implant soft tissue and potentially contributing to a pathologic foreign body reaction.

**Figure 6:**
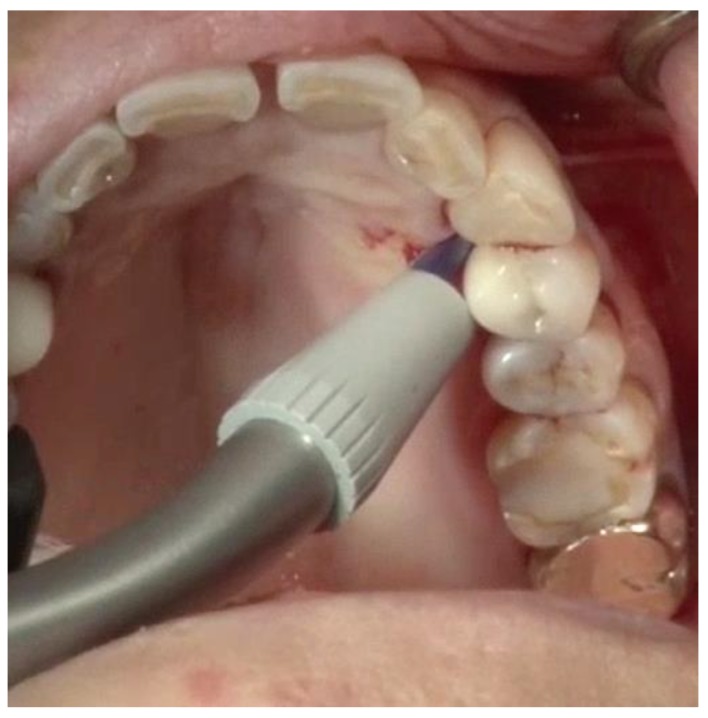
The tip of the retractor on the videoscope is rotated to an optimal angle and used to gently push back the lingual flap to allow visualization of the bone loss.

The incisions used are very similar to those previously described for VMIS around natural teeth. A sulcular incision is made around the implant in the area of bone loss. No soft tissue is removed at this point. The incision is extended apically to the level of remaining bone. A split-thickness incision is then made across the base of the adjoining papilla extending to the line angle of the adjacent teeth. As with natural teeth, the split-thickness incision is extended apically for an adequate distance to allow for the insertion of the retractor tip on the videoscope and to allow adequate visualization of the bone loss. (Figure 6). 

**Figure 7:**
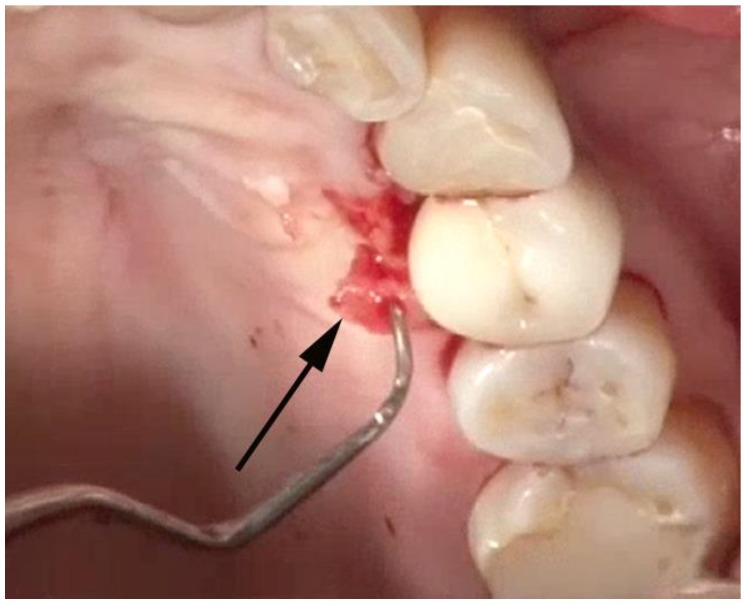
A thin (1–2 mm) section of tissue (arrow) containing foreign particles of titanium or cement is removed from the area of bone loss.

Once the implant has been adequately visualized, under videoscope guidance and using a new small blade, a thin section of tissue approximately 1–2 mm in thickness is removed from the tissue that was in contact with the implant in the area of bone loss. (Figure 7) When this tissue is evaluated histologically, as was done in the study by Wilson et al., multiple areas of cement and/or titanium particles surrounded by inflammatory cells are often noted. The purpose of removing this tissue is to eliminate foreign material that may cause further damage to the implant-supporting bone in the future as well as may interfere with the regeneration of bone around the implant.

**Figure 8:**
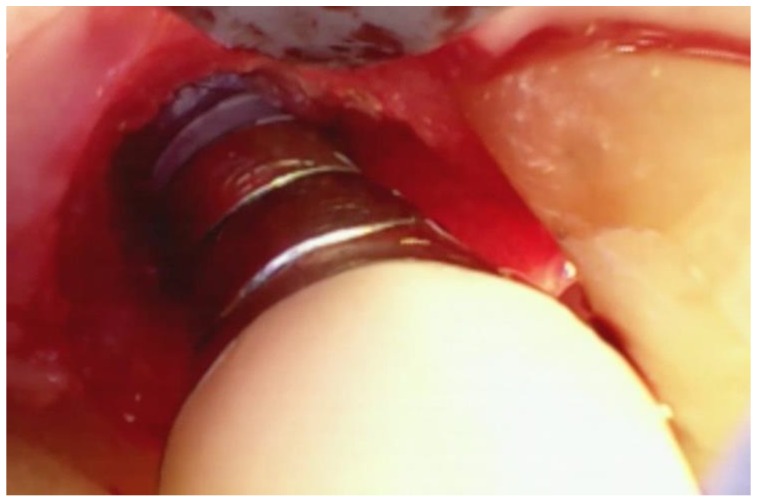
The fully debrided bony lesion can be visualized after removal of the granulation tissue.

Following the removal of the thin section of tissue, any granulation tissue is removed in a similar manner as performed on natural teeth. However, care should be taken to not touch the implant surface with the instrument used to remove the granulation tissue. The implant surface is covered by titanium oxide, and this has been shown to be an integral part of the osseointegration process [22]. Titanium oxide is very fragile and will be damaged or corroded if the implant surface is touched by an instrument or disinfected with harsh chemicals such as citric acid or a tetracycline solution. Because of this, great care is taken to not touch the implant with a curette while removing the granulation tissue and to only gently wipe the implant surface with sterile gauze soaked in saline. The debrided peri-implant bone loss is shown in Figure 8.

**Figure 9:**
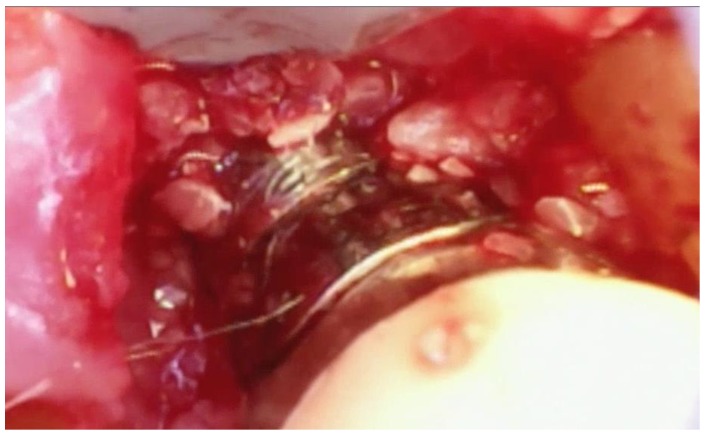
The bony lesion pictured in Figure 8 has been filled with particulate bone-grafting material mixed with EMD.

After the lesion is debrided of granulation tissue and the implant is cleaned with saline, DFDBA mixed with EMD is place in the bony defect. (Figure 9). 

EDTA is not burnished on the surface of the implant. Radiographs taken one year post-surgery appear to show bone formation in the area of initial bone loss. Further, it appears that the new bone has formed in contact with the implant surface. The frequently seen “black line” between the implant and newly formed bone is not present. The lack of a black line may be an indication of re-osseointegration. However, this can only be proven with a block biopsy of the implant and bone. This type of biopsy is not ethically acceptable with a clinically functional implant, so the existence of re-osseointegration remains a conjecture.

## 5. Summary

**Figure 10:**
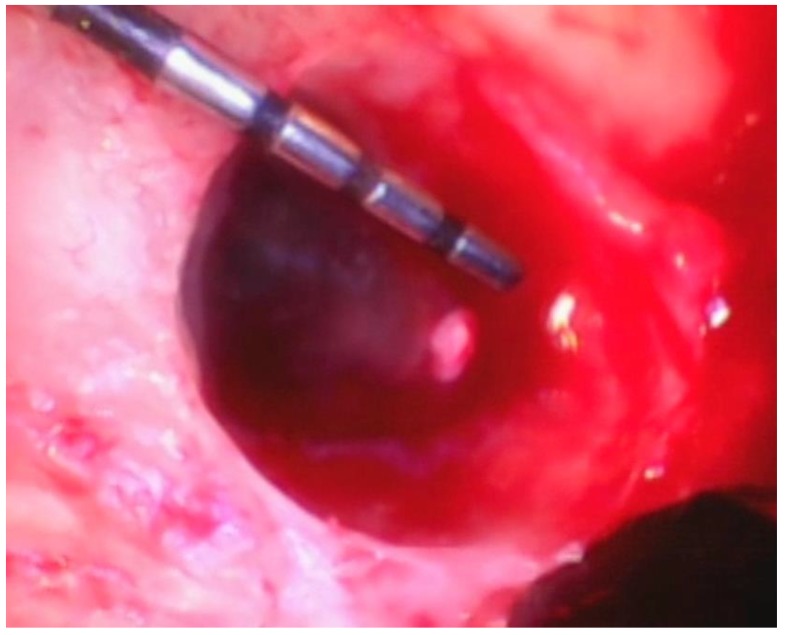
Videoscope visualization of the apex of a maxillary incisor during surgical endodontic treatment.

**Figure 11:**
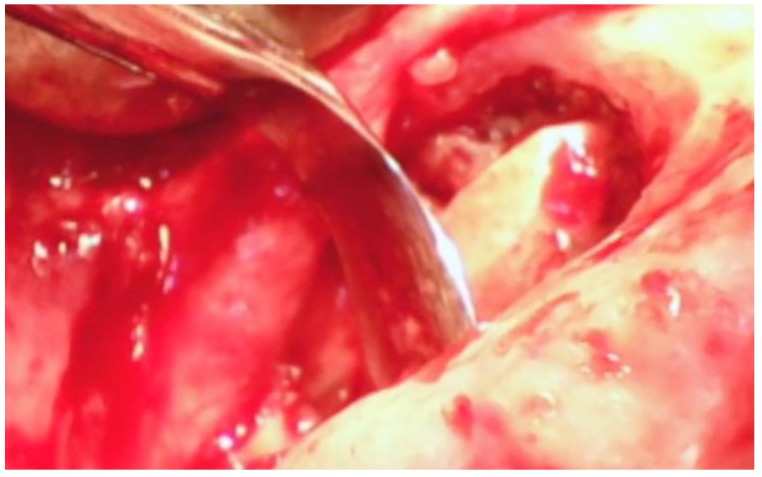
Videoscope visualization of a lateral window access for sinus bone grafting in preparation for placing implants.

The existing literature on minimally invasive periodontal surgery, the literature on the videoscope, the literature on VMIS, and the techniques designed around the advantages of the videoscope have been reviewed. The published clinical results are favorable and indicate that VMIS can be used to consistently regenerate bone around periodontally damaged teeth with no recession and a possible improvement in soft tissue height. In addition, the application of the VMIS technique to peri-implant bone loss has been reviewed. The videoscope is also being used for other surgical and non-surgical procedures. Some of these are surgical and non-surgical endodontic procedures (Figure 10), sinus elevation surgery (Figure 11), and non-surgical hygiene procedures. Research in these applications is ongoing. 

Patents—Dr. Harrel holds the patent for the videoscope used in VMIS.
